# Hierarchical Structure of the *Cocos nucifera* (Coconut) Endocarp: Functional Morphology and its Influence on Fracture Toughness

**DOI:** 10.3390/molecules25010223

**Published:** 2020-01-06

**Authors:** Stefanie Schmier, Naoe Hosoda, Thomas Speck

**Affiliations:** 1 Germany and Freiburg Center for Interactive Materials and Bioinspired Technologies (FIT), Georges-Köhler-Allee 105, D-79110 Freiburg, Germany; 2 Plant Biomechanics Group, Botanic Garden, Faculty of Biology, University of Freiburg, Schänzlestraße 1, D-79104 Freiburg, Germany; 3 National Institute for Materials Science, Namiki, Tsukuba 305-0044 1-1, Japan; hosoda.naoe@nims.go.jp; 4 Germany and Cluster of Excellence *liv*MatS @ FIT, Freiburg Center for Interactive Materials and Bioinspired Technologies, Georges-Köhler-Allee 105, D-79110 Freiburg, Germany

**Keywords:** *Cocos nucifera*, coconut endocarp, hierarchical structure, functional morphology, fracture toughening mechanisms

## Abstract

In recent years, the biomimetic potential of lignified or partially lignified fruit pericarps has moved into focus. For the transfer of functional principles into biomimetic applications, a profound understanding of the structural composition of the role models is important. The aim of this study was to qualitatively analyze and visualize the functional morphology of the coconut endocarp on several hierarchical levels, and to use these findings for a more precise evaluation of the toughening mechanisms in the endocarp. Eight hierarchical levels of the ripe coconut fruit were identified using different imaging techniques, including light and scanning electron microscopy as well as micro-computer-tomography. These range from the organ level of the fruit (H0) to the molecular composition (H7) of the endocarp components. A special focus was laid on the hierarchical levels of the endocarp (H3–H6). This investigation confirmed that all hierarchical levels influence the crack development in different ways and thus contribute to the pronounced fracture toughness of the coconut endocarp. By providing relevant morphological parameters at each hierarchical level with the associated toughening mechanisms, this lays the basis for transferring those properties into biomimetic technical applications.

## 1. Introduction

The hierarchical architecture of plant materials is well known and can evoke remarkable mechanical properties that are also useful for technical materials [1,2,3,4]. An important element for understanding the mechanics of woody tissue is the cell wall, which is essentially made up of only four groups of macro-molecules (cellulose, hemicellulose, pectin, and lignin). By varying the amount and arrangement of these molecules in the cell wall and by altering the cell shapes and tissue arrangements, a broad spectrum of mechanical properties has evolved [2]. Hierarchical architecture describes the structural variations that take place on different length scales, starting from the molecular structure of individual cell components, the cellular structure of tissues, up to the arrangement of different tissues in plant organs. At each level, individual parameters can be set, which, in combination, influence the mechanical properties of the entire plant or plant organ.

In recent years, the biomimetic potential of lignified or partially lignified fruit pericarps and seed coats as concept generators for puncture resistant and local impact energy dissipating materials systems has been analyzed (*Acrocomia mexicana* endocarp [5]; *Cocos nucifera* endocarp (e.g., [6,7,8,9]); *Macadamia integrifolia* seed coat (e.g., [4,10,11]); *Orbignya speciosa* endocarp [12]). The macadamia seed coat has been particularly well studied in this context. Schüler et al. [11] were able to identify that the macadamia seed coat is highly anisotropic and comprises nine hierarchical levels. Several of these hierarchical levels influence the crack paths and thus have a great influence on the mechanical behavior of the entire seed coat. For the cocoyol palm endocarp (*Acrocomia mexicana*), Flores-Johnson et al. [5] identified two different layers and a radial decrease of density in the material from the outside (mesocarp to the inside (testa)), as well as concomitantly a decreasing elastic modulus and hardness.

The fruit of *Cocos nucifera* is a drupe in which only the endocarp, the innermost layer of the pericarp, comprises a massive and solid lignified structure. A characteristic for the outer shape of the coconut endocarp is the three longitudinal ridges, which are formed during growth by the fusion of the three carpels. Three pores, the micropyles, are visible at the basal end between the ridges. During sprouting, the seedling grows out through one of these pores, which is—in contrast to the other two pores—not lignified [13,14,15,16]. Microscopic studies by Winton [13] on mature fruits note that the endocarp consists of stone cells and vascular bundles, in which only the xylem elements are visible due to tissue rupture during growth and the presence of fungal hyphae. Additionally, he identified spiral and pitted tracheae as xylem elements.

The aim of the present study is to analyze and visualize the functional anatomy, i.e., the form-structure-function relationship, of the coconut endocarp on multiple hierarchical levels, ranging from the entire fruit to the sub-cellular level. For this purpose, three imaging techniques—computer tomography (CT), light microscopy (LM), and scanning electron microscopy (SEM)—were applied to ensure the appropriate resolution for each hierarchical level. Finally, these findings were used to more precisely evaluate the toughening mechanisms of the coconut endocarp in the identified hierarchical levels.

## 2. Results

**Figure 1:**
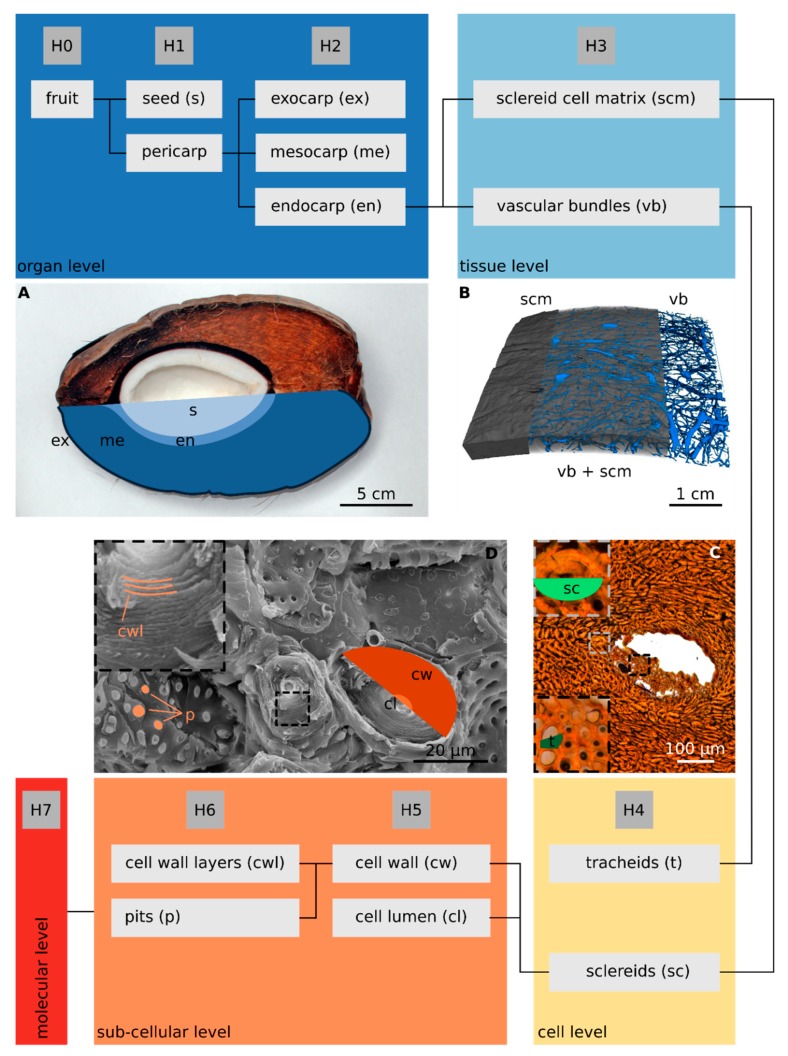
Hierarchical levels of the coconut fruit with illustrations of the important parameters of each level. (**A**) Photograph of an entire fruit cut in half in a longitudinal direction. (**B**) Computer tomography (CT)-reconstruction of an endocarp sample (scan resolution: 42 µm). (**C**) Light microscopy (LM) image of a polished thin section of the endocarp showing the cross section of a vascular bundle in the sclereid cell matrix. (**D**) SEM micrograph of a fractured endocarp surface showing details of the sclereid cell matrix. Table 1 provides the corresponding morphological parameters. H0–H7: Eight hierarchical levels; cl: Cell lumen, cw: Cell wall, cwl: Cell wall layers, en: Endocarp, ex: Exocarp, me: Mesocarp, p: Pits, s: Seed, sc: Sclereids, scm: Sclereid matrix, t: Tracheids, vb: Vascular bundles.

For reasons of comparability, the hierarchical classification employed here has been adapted to the scheme of Schüler et al. [11], developed for the macadamia nut and seed shell. In the present study, eight hierarchical levels of the coconut fruit could be identified (Figure 1), of which the various levels of the endocarp were examined in more detail (Figure 1 H2–H6). The characteristic parameters for each hierarchical level are summarized in Table 1. Furthermore, the description of the coarsest hierarchical levels (H0–H2) can be found in the literature, e.g., as cited in Table 1, or studied on the basis of the CT reconstruction of the entire coconut fruit in the Supplementary Material, which also shows the hierarchal levels H0–H2 [Supplementary Information Movie S1].

### 2.1. Hierarchical Level H2—Endocarp

The endocarp, which represents the innermost part of the three-layered coconut fruit wall, has a prolate, spheroidal shape, with the long axis in the tested samples with a mean diameter of 116.5 ± 9.2 mm and the short axis a mean diameter of 92.8 ± 5.2 mm. The ends of the long axis have the largest curvature. At one end of the endocarp, three micropyles (germinal pores) are located between the three ridges running around the longitudinal axis of the endocarp. The ridges originate from the ontogenetic fusion of three carpels. Additionally, the thickness of the endocarp was very variable with differences of 54.7% (±11.4%). The thickest region (4.7 ± 0.6 mm) is located near the three micropyles, and the thinnest region (2.5 ± 0.5 mm) situated on the opposite side of the endocarp.

### 2.2. Hierarchical Level H3

**Figure 2:**
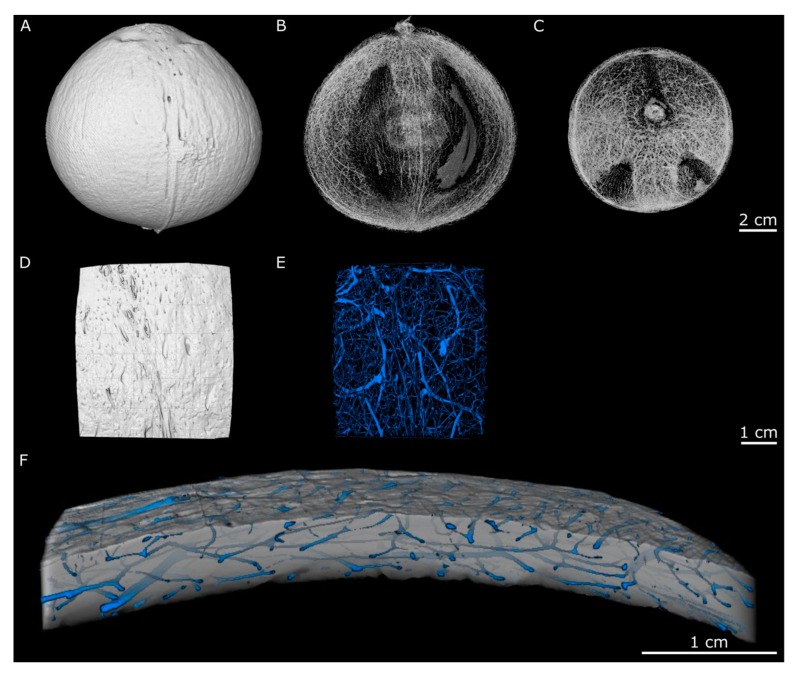
µCT reconstructions of a coconut fruit scanned at two resolutions: 150 µm (**A**–**C**) and 42 µm (**D**–**F**). Side view of the coconut endocarp showing the outer surface (**A**) and the course of the vascular bundles (**B**). Top view of the endocarp with highlighted vascular bundles (**C**). Top view of a smaller endocarp volume scanned at higher resolution showing the outer surface (**D**) and the course of the vascular bundles (**E**). The higher magnification also reveals smaller vascular bundles, which span a strongly branched network between the larger vascular bundles. In the enlarged side view of the smaller endocarp volume, the vascular bundles are observed running almost parallel to the outer surface of the endocarp (**F**). Images (**B**,**C**) are kindly provided by the Shimadzu Corporation.

At the tissue level, the endocarp shows a complex structure consisting of a dense matrix material with an integrated 3D-network of less dense channels, representing (the remains of) the vascular bundles (Figure 2). In the coarse CT reconstruction (scan resolution: 105 µm) of the entire endocarp (Figure 2A), the vascular bundles are observed to run mainly along the boundaries of the three carpels. In the reconstruction of a more detailed scan (scan resolution: 42 µm), vascular bundles with a smaller diameter become visible and reveal a highly connected network covering the entire endocarp (Figure 2B). The reconstruction analysis with the highest resolution (42 µm) reveals a diameter of 0.22 ± 0.14 mm for the vascular bundles. Due to the network of vascular bundles, the matrix material has a porosity of 3.3% as determined from CT measurements.

### 2.3. Hierarchical Level H4

**Figure 3:**
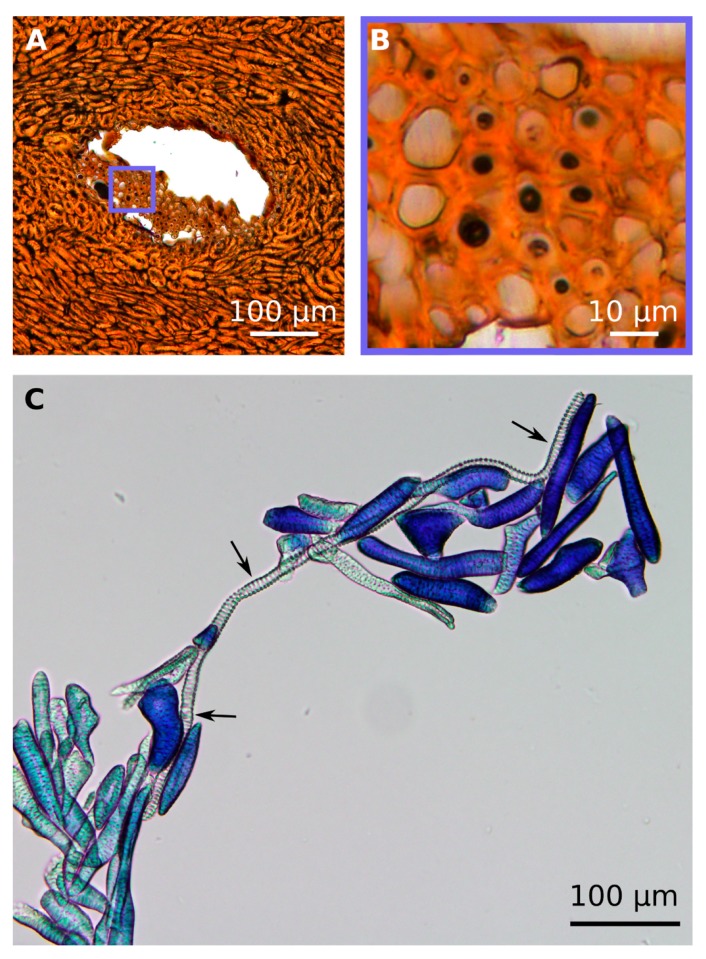
LM images from the coconut endocarp showing tracheids. In the cross section of a vascular bundle ((**A**), polished thin section), the polygonal shape of tracheid cross-sections is visible (**B**). Macerated endocarp cells stained with Toluidin blue, showing an intact tracheid cell (⟶) with a length of 627 µm between accumulated sclereid cells (**C**).

In the mature coconut endocarp, only the xylem elements of the vascular bundles are visible. The xylem elements are considered to be tracheids, due to their dimensions (small diameter) and the lack of perforation plates, which are typically found in vessels. The xylem elements have a diameter of 11.5 ± 3.6 µm and a length of 424.3 ± 263.3 µm (Figure 3).

**Figure 4:**
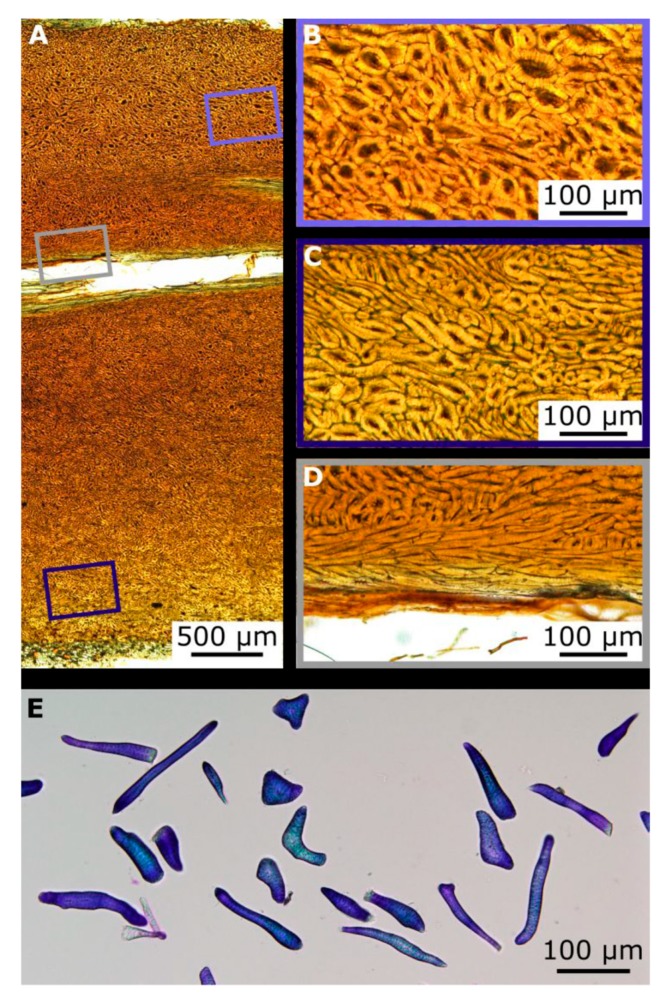
LM images from the coconut endocarp showing sclereids. The cross section of a polished thin section shows a longitudinally cut vascular bundle within the brown tissue of the sclereid cells (**A**). Higher magnifications of the sclereid cell matrix reveal larger cells near the mesocarp side (**B**) than near the testa (**C**). Very long sclereids, the sclerenchyma fibers, are located in parallel to the vascular bundles (**D**). Macerated endocarp cells stained with Toluidin blue, showing sclereid cells with various shapes (**E**).

**Figure 5:**
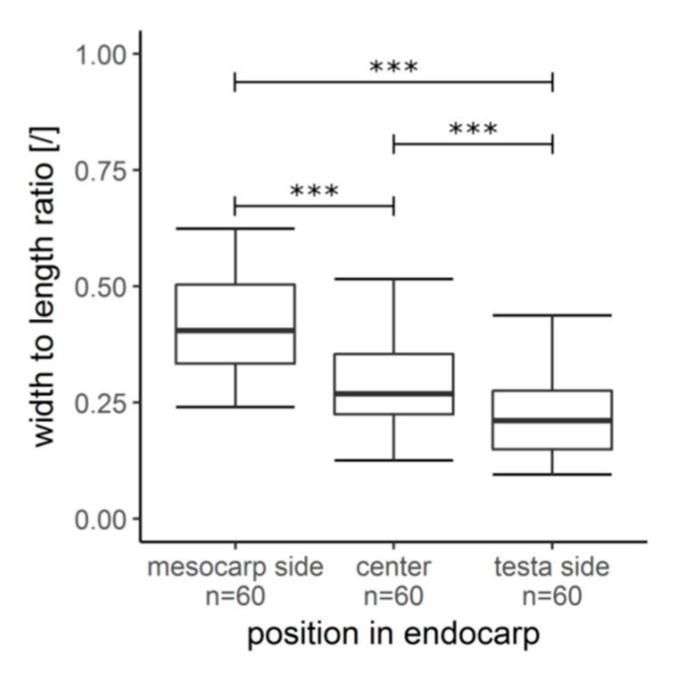
Comparison of the width-to-length ratio of the sclereid cells along the endocarp cross section. It was observed that the width-to-length ratio decreased significantly from the mesocarp side to the testa side (one-way analysis of variance, F (2177) = 68.91, *p* < 0.001 (***), Tukey HSD post-hoc testing).

The sclereid cell matrix consists of stone cells (roundish sclereid cells) and (slightly) elongated sclereid fibres, of which the fibres are mainly arranged parallel to the vascular bundles and often adjacent to the sclerenchyma caps. The shape of the sclereid cells varied greatly (Figure 4), but it was observed that their length increases from the mesocarp side to the testa, whilst their width decreases (Table 1). A one-way ANOVA revealed a significant decrease in the width to length ratio between the three regions along the cross section (Figure 5).

### 2.4. Hierarchical Level H5

**Figure 6:**
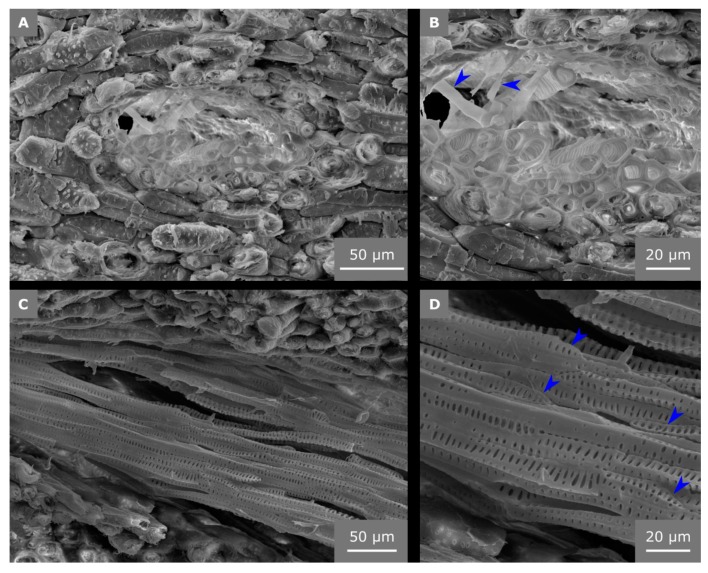
SEM images of vascular bundles from fracture surfaces. In the cross section, only the tracheids are visible in the mature endocarp (**A**) and have a polygonal cross section (**B**). From the former phloem cells, only fragments are left (arrows in (**B**)). In a longitudinally broken vascular bundle (**C**), scalariform thickenings of the tracheids are clearly visible (**D**). No pronounced perforation plates are visible at the ends of the tracheids (arrows in (**D**)).

As pointed out in Section 2.3, in the vascular bundles found in the mature endocarp, only the xylem elements remain clearly visible as cellular structure (Figure 6A,B). The phloem cells, which transported assimilates as nutrients to the growing young coconut fruit in earlier ontogenetic stages, are barely visible in the mature endocarp and persist only as small broken structures (Figure 6B). The tracheids have a polygonal cross section (Figure 6A,B), and the thickness of the tracheid cell wall is 1.7 ± 0.4 µm. The tracheid cells show scalariform pitting with oval bordered pits, which covers the major part of their cell walls and increases their bending and tension stiffness (Figure 6C,D).

**Figure 7:**
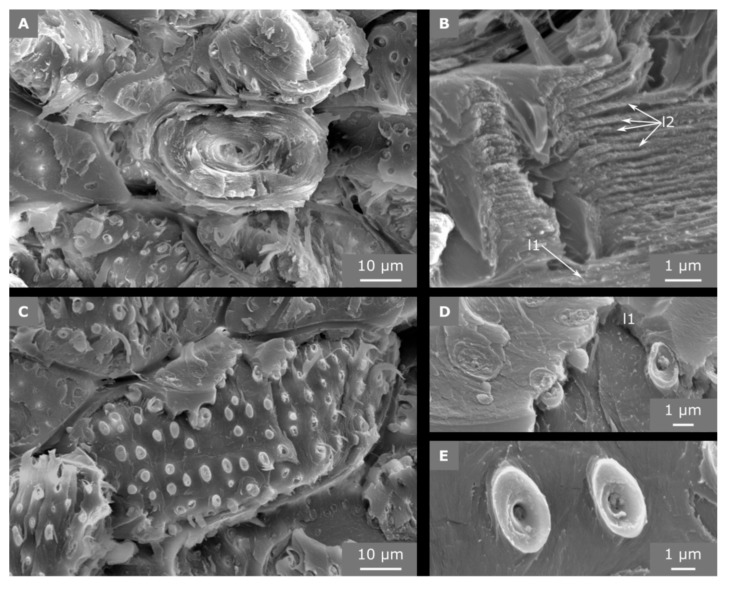
SEM images of sclereid cells from fracture surfaces. The cross-section shows the many cell wall layers that fill almost the entire cell lumen (**A**). The cell wall is traversed by pit canals (**B**), to which the lignified secondary cell wall layers (l2) align. In a sclereid cell oriented longitudinally to the direction of fracture, the crack has run into the cell wall and detached the outermost cell wall layers (**C**). When the primary cell wall (l1) and middle lamella is partly broken, the pit cavity becomes visible (**D**) and the bordered pits emerge from the fracture surface (**E**).

The vascular bundles are embedded in a matrix of stone cells (roundish sclereid cells) and (slightly) elongated sclereid fibers. The sclereid cells have a round cross section and their cell walls fill the cell lumens almost completely (Figure 7). The thickness of the entire cell wall is 89.1 ± 4.2% of the cell radius, which leads to an area fraction of the cell wall of 98.8% of the entire cell cross-sectional area.

### 2.5. Hierarchical Level H6

**Figure 8:**
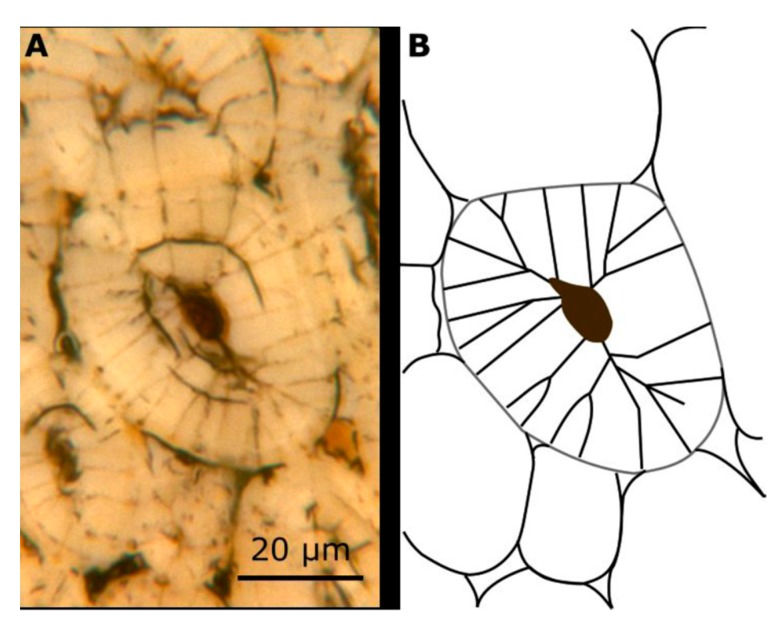
LM image (**A**) and schematic drawing (**B**) of the pit canal system of a sclereid cell.

The finest hierarchical level presented in this study is the cell wall structure of the sclereid cells. The primary cell wall (including half of the middle lamella) has a thickness of approximately 2.0 ± 0.5 µm and is significantly thicker compared to the individual secondary cell wall layers with a thickness of 253 ± 74 nm, which are comprised of various layers (>30) and form pit canals (Figure 7B).The secondary cell wall layers are deposited consecutively one after the other from the living cell body in the center of the sclereid cells, so that the innermost secondary cell wall layer is the last deposited (youngest) layer. On fracture surfaces, these bordered, ramiform pits (Figure 8) appear like small chimneys emerging from the surface (Figure 7D,E), giving it a structural roughness.

**Figure 9:**
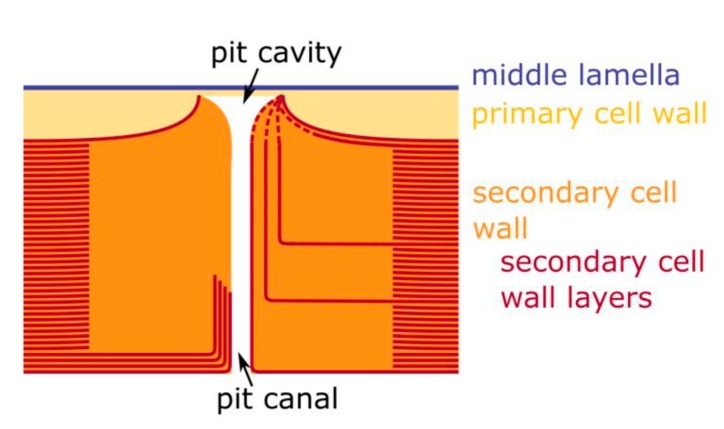
Diagram of a bordered pit. Left side of the pit cavity: Observed structural features. Right side of the pit cavity: Hypothesized structuring in the vicinity of the pit cavity (for reasons of clarity not all individual cell wall layers-red lines- are traced to the pit cavity).

Individual fracture surfaces of the sclereid cells reveal a conspicuous pit structure: (1) Secondary cell wall layers of pit canal segments run in parallel to the pit canal (Figure 7B, lower part of the canal). (2) The pit protrudes and becomes visible as tiny chimneys if the primary cell wall layer is removed by the fracture process (Figure 7D,E). (3) The detailed structuring of the secondary cell wall layers, however, remains unclear at the border of the pit cavity and must result from developmental processes, which could not be observed. However, it appears as if individual secondary cell wall layers merge or become thinner at the rim of the pit cavity, where secondary cell wall layers are in contact with the primary cell wall (Figure 9).

## 3. Discussion

The hierarchical architecture of the coconut endocarp could be delimited to six hierarchical levels, which were visualized by the use of three imaging techniques (CT, LM, and SEM). A common feature of the hard shells of coconut, cocoyol, and macadamia is that the outermost sclereid layer consists of nearly isodiametric sclereids as can be seen for the coconut in Figure 4 [5,11]. In its general structure, the coconut endocarp more resembles the cocoyol endocarp [5] than the multi-layered macadamia seed coat, which represents a biological micro-laminate [11]. As for the cocoyol endocarp, the sclereid cells of the coconut endocarp have larger diameters and are more isodiametric on the mesocarp side compared to the sclereids on the testa side, which are more elongated (Figure 4A). As already described by Winton [13], the longitudinal axes of the sclereids are always oriented parallel to the outer surface of the endocarp (Figure 4B,C), with only the sclerenchyma fibers of the sclerenchyma cap aligned to the vascular bundles differing from this orientation. In contrast, however, to the cocoyol fruit, we found a continuously decreasing width-to-length ratio for the sclereids from the mesocarp side to the testa side. Therefore, we considered the sclereid cell matrix as one layer that builds up the entire endocarp, but with a gradient in cell size from the outer mesocarp side to the inner testa side (Figure 5). An additional difference is the endocarp thickness, which is quite constant for the cocoyol fruit (3–4 mm) [5], though varies by up to 50% for the coconut fruit. Furthermore, a characteristic structural element of the coconut endocarp is the branched network of vascular bundles.

Unfortunately, Flores-Johnson et al. [5] did not give any details about the appearance of the vascular bundles in the cocoyol endocarp. Similar to the descriptions of Winton [13], the tissue of the vascular bundles was already quite degenerated in coconut samples use for the present study, e.g., fungal hyphae were found in some vascular bundles within the embedded samples. Only one cell type could be detected, which we characterized due to its structural feature (cell dimensions and missing perforation plates) as tracheids with scalariform pitting (Figure 6). Although vessels were found in roots, stems, and leaves of *Cocos nucifera* [17], they seem not to occur in the endocarp. The diameters of vessels found in leaves are with 180–230 µm ca. 10 times larger than those measured in this study for the endocarp tracheids (11.5 ± 3.6 µm). In addition, we could not detect any vessel elements (neither in the LM investigations of macerated cells, nor in the SEM investigations), which are a characteristic of vessels [18]. Nevertheless, the magnitudes of the structural elements of the coconut endocarp measured by Gludovatz et al. [7] are in accordance with our results. They named the vascular bundles “hollow channels”, the tracheids “hollow fibers”, and the sclereids “hollow cells”, but did not describe any further structuring of the cell wall, whereas we could show that the cell wall of the sclereid cells is composed of individual layers and traversed by pit canals (H6) as shown in Figure 7.

Our results allow a hypothesis concerning the structuring of the pits, which are formed by secondary cell wall layers that seem to run in parallel to the pit canal (Figure 9). Although the cell wall structure at the border of the pit cavity is not yet entirely clear, this hypothesis fits observations about the general structure of bordered pit [18] and findings for the pits of vascular parenchyma cells with secondary cell walls of another monocotyledonous plant (*Dracaena draco*) [19].

Finally, the fracture surfaces and the observations of Gludovatz et al. [7] were used to hypothesize about the influence of the hierarchical structure on the formation and development of cracks, i.e., on the toughening mechanics of the coconut endocarp. Following on from how Van Mier and Man [20] have described the fracture of concrete and other disordered materials, the coconut endocarp can be ascribed similarly due to its high density of aggregates (sclereids) and small amount of matrix material (middle lamella). Within such materials, micro-cracking occurs in the interfacial transition zone between the matrix and aggregates in the loading direction [20]. The stone cells are arranged with their longitudinal axes parallel to the outside, and due to the ellipsoidal shape of the endocarp, their longitudinal axes are always perpendicular to the loading direction. Micro-cracks, therefore, probably occur along their short axes, which are considerably smaller than the critical crack length of 78 µm (calculated with the Griffith equation and using the critical stress intensity factor of 3.2 MPa m^1/2^ [7], the critical strength of 205 MPa [9], and a geometrical constant of Y = π). This essentially means that the (micro-)structuring of the endocarp prevents a critical crack length from being reached during micro-crack formation alone, but that individual micro-cracks must join to reach a critical length, which can be considered as a structural feature that increases toughness and reduces the probability of breaking.

After a macro-crack has developed, toughening mechanisms complicate the progress of crack opening. Ritchie [21] described the concept of extrinsic and intrinsic toughening mechanisms, which Wegst et al. [1] considered as an essential factor for the damage tolerance of natural structural materials, such as bone or nacre. Both mechanisms inhibit crack growth, with the intrinsic processes acting in front of the crack tip at both nano- and microscales to restrict crack propagation, whilst the extrinsic factors behind the crack tip act at higher length scales to reduce local stresses/strains [1].

**Figure 10:**
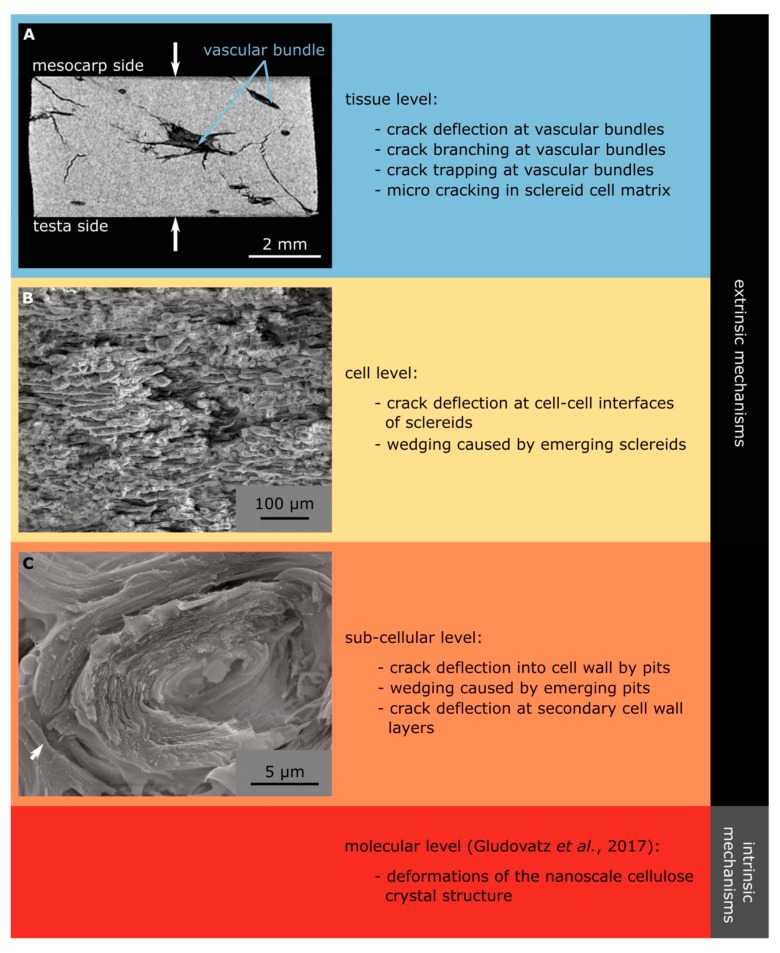
Extrinsic and intrinsic toughening mechanisms found in the coconut endocarp. µCT reconstruction of a fractured sample in cross section; sample tested in compression in the study of Lauer et al. [9] (**A**). SEM images showing details of the tortuous crack path in the sclereid cell matrix (**B**) and in a single sclereid cell (**C**).

**Figure 11:**
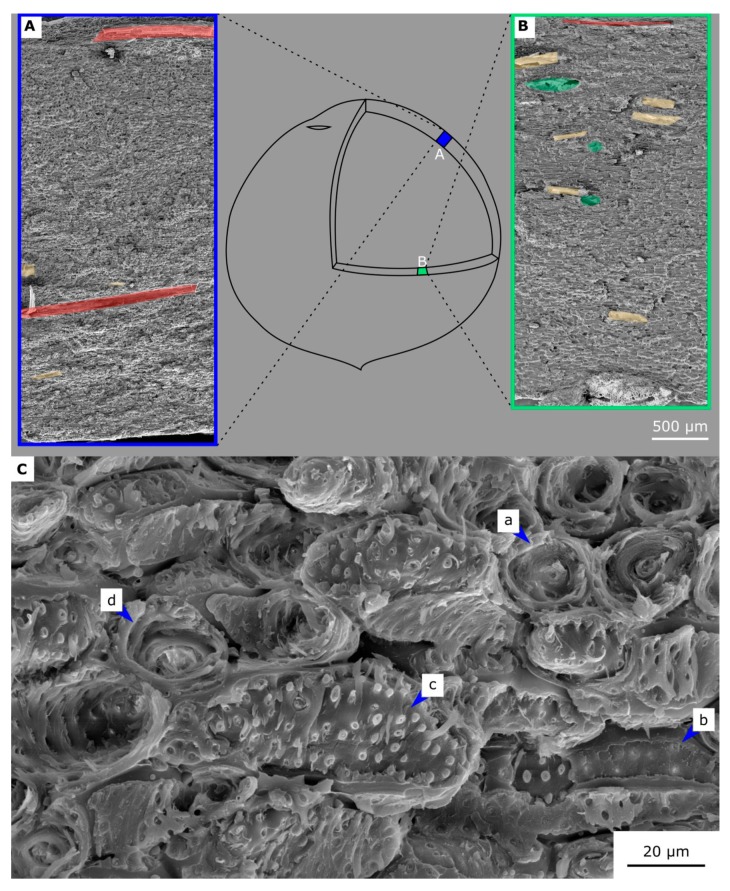
SEM images of fracture surfaces broken in meridional/sagittal plane (**A**) and equatorial/transverse plane (**B**). Vascular bundles parallel to the fracture surface are marked red, the perpendicular ones are green, and the oblique ones are yellow. The detail of a broken sclereid cell matrix (**C**) highlights the four failure modes (modified after [5]: Cell tearing (a), middle lamella breakage (b), cell wall breakage (c), and pull-out of elongated cells (d).

In the following, this concept is used to describe the fracture behavior of the coconut endocarp at the different hierarchical levels (Figure 10). The mechanical properties of the coconut endocarp are anisotropic as already shown by Schmier et al. [22] and Gludovatz et al. [7]. One of the structural elements influencing this behavior is most probably the anisotropic network of vascular bundles, which traverse the sclereid matrix (Figure 2) [7]. Fracture surfaces broken in the meridional plane (Figure 11A) and in the equatorial plane (Figure 11B) differ in the way that the vascular bundles cross the fracture surface. In the meridional plane, there are fewer vascular bundles present and they are usually broken lengthwise, while in the equatorially broken surface, there are more vascular bundles that are usually broken perpendicularly. The vascular bundles contribute to the extrinsic toughening mechanisms leading to crack deflection, crack branching, and crack trapping (Figure 10A), which is also stated by Gludovatz et al. [7]. In addition, the images of a crack tip from this study show that micro-cracks occur in front of the (macro-)crack tip in the sclereid cell matrix.

Extrinsic toughening mechanisms are also found in the sclereid cell matrix. Flores-Johnson et al. [5] described four failure modes for the sclereids of the cocoyl endocarp: Cell tearing (a), middle lamella breakage (b), primary cell wall breakage (c), and pull-out of elongated cells (d). These failure modes were also present in the sclereid cell matrix of the coconut endocarp (Figure 11C). In the case of the coconut, we found that failure mode (c) is not only a fracture of the primary cell wall, but also a partial failure of (some of) the secondary cell wall layers (Figure 11C). Therefore, we generalize that this failure mode is due to cell wall breakage, including primary and (parts of the) secondary cell wall. In the sclereid cell matrix, crack redirection is also one of the extrinsic factors (now at the cellular level and not at the tissue level) as the crack is redirected between the sclereid cells (failure mode (b)), following in its path the middle lamellas. Furthermore, wedging caused by emerging sclereids creates additional frictional resistance, which reduces the forces at the crack tip.

The sub-cellular structure of the sclereids also contributes to the toughness of the endocarp, with important structural elements being the pits and the multi-layered structure of the cell wall, as well as the finding that neighboring sclereid cells are closely connected at the pit pairs. It is therefore likely that the pits help to prevent the crack from running between the cells and force it to penetrate into the cell wall of one of the cells (failure modes (a), (c), and (d), arrow in Figure 10C). Due to the multi-layered structure of the cell wall, the tip of the crack is blunted repeatedly when the individual cell wall layers are fractured. From the fracture surfaces presented by Flores-Johnson et al. [5], we expect that similar pits are also present in the cocoyol endocarp. The pits might toughen the endocarp further by protruding from the fracture surface and thus increase the crack surface roughness and contributing to wedging. This wedging might also explain the additional increase in force during compression tests when a sample already shows dominant cracks, as reported by Lauer et al. [9]. As an intrinsic toughening mechanism, Gludovatz et al. [7] reported deformations of the nanoscale cellulose crystal structure, which were not studied here.

## 4. Materials and Methods

A ripe, dried, whole coconut was purchased online (Japan Ornaments and Artificial Flowers Co., LTD., Tokyo, Japan) for CT analysis, which was performed in Japan. Additionally, 10 ripe coconut fruits, with their exo-and mesocarps already removed, were purchased from a local supplier (Greenyard Fresh Germany GmbH, Freiburg, Germany) and stored at ambient conditions until examination for the other analyses carried out in Germany.

### 4.1. Sample Preparation

The lengths and widths of the 10 coconut endocarp samples without exo-and mesocarps were measured using a manual calliper. The fruits were cut open in a longitudinal direction using a band saw and the endosperm was removed. To cope with the thickness variation of the endocarp, the thickest and thinnest regions of the cut surface were also determined using a digital calliper (SMT023, AGT^TM^) with an accuracy of 10 µm.

### 4.2. CT Measurements

The dried whole coconut was scanned in a micro-focus x-ray CT-system (inspeXio SMX-225CT FPD HR, Shimadzu Corporation, Tokyo, Japan) using three magnifications (scan resolution: 208 µm, 150 µm, 42 µm). Further data processing to determine the diameter ranges of the vascular bundles and the porosity of the sclereid cell matrix was carried out with CT Analyser V.1.18.4.0 (Bruker microCT, Kontich, Belgium). Visualization of the data was performed with Avizo (V 9.2.0, FEI Company, Hillsboro, OR, USA).

### 4.3. Light Microscopy

Small plates were cut from three endocarp samples with a precision cutter (MICRACUT 151, Metkon Instruments Inc., Osmangazi, Turkey) with the following dimensions: Length = natural thickness of endocarp (approximately 3.7 mm), width = 2.5 mm, thickness = 270 µm. Subsequently, the lengths of these plates were divided into three equal-sized platelets with a razor blade according to the mesocarp side, center, and testa side. The platelets were placed for seven days in 50% bleach (Eau de Javel, Floreal Haagen GmbH, Wadgassen, Germany) for maceration and afterwards put for another day in distilled water. Toluidine blue stain was used to increase the contrast for light microscopy investigation. A drop of the staining solution was placed on a slide and a platelet was laid on top and covered with a cover glass. Light pressure was applied by hand to the cover glass and it was sheared slightly to separate the cells. After an exposure time of 1.5 min, the dye was washed out with water and filter paper. In addition, cut out pieces of the endocarp were embedded in acryl [Appendix A], cut into thin sections with a precision cutter, and sequentially ground on silicon carbide grinding paper (#800–#4000, Struers A/S, Ballerup, Denmark) to a grain size of 5 µm using a micro grinding machine (EXAKT 400 CS, EXAKT Advanced Technologies GmbH, Norderstedt, Germany). All samples were then examined with the light microscope (BX61, Olympus, Tokyo, Japan), equipped with a digital camera (DP71, Olympus, Tokyo, Japan), using the cellP software V.2.8 (Olympus, Tokyo, Japan). The pictures were then analyzed using ImageJ V.1.52c [23]. The dimensions of the tracheids were determined using the segmented line tool. Additionally, the dimensions of the sclereids were approximated with a polygon, and Feret’s diameters were used to measure cell widths and lengths.

### 4.4. Scanning Electron Microscopy

Fracture surfaces were produced using a hammer. The samples were fixed to aluminum stubs with conductive adhesive pads (G3347, Plano GmbH, Wetzlar, Germany), gold-sputtered (Sputter Coater 108 auto, Cressington Scientific Instruments Ltd., Watford, UK), and investigated with an Scios HiVac 2 (Thermo Fisher Scientific, Waltham, MA, USA).

### 4.5. Statistics

Data processing was conducted with the free software GNU R V.3.4.0 [24]; for statistical testing and plotting of the data, the additional packages car [25] and ggplot2 [26] were applied. The data were checked for normal distribution (Shapiro–Wilk test) and variance homogeneity (Levene test). To test for differences between the three groups, the data were subjected to a one-way analysis of variance, followed by a Tukey HSD post-hoc test.

## 5. Conclusions

In the present study, we have analyzed the hierarchical architecture of the coconut endocarp, which comprises six hierarchical levels, covering length scales over 10 orders of magnitude from 0.1 m for the intact endocarp to the individual cell wall layers on the sub-cellular level (in the 10–100 nm range). Using three imaging techniques (CT, LM, and SEM), we were able to describe the structural composition of the endocarp in much detail. We could show that the 3D-network of vascular bundles consists of scalariform pitted tracheids (the phloem elements are barely visible in the mature endocarp) and plays an important role for the integrity of the endocarp structure, which can be interpreted as fiber-reinforced composite with a sclereid matrix showing a size and form gradient of the constituent sclereid cells. In addition, we detected a further structural element, the lignified pits, which link in a similar way as dowels neighboring sclereid cells and, by this, increase the structural integrity and toughness of the endocarp matrix. After micro-fracturing, the protruding remains of the pits markedly increase the surface roughness and friction within a micro-crack and contribute so further to the toughening of the endocarp. We consider these pit structures as highly relevant for the fracture behavior of the coconut endocarp and probably also for other lignified fruit shells and seed coats. Based on the detailed analysis of the hierarchical structuring of intact and broken endocarp samples, we have been able to link the results of the functional anatomical investigations with the fracture mechanical behavior of the coconut endocarp, detecting toughening mechanisms at each hierarchical level.

Coconut endocarp accumulates in large amounts during the “harvest” of coconut flesh (copra) and coconut milk. It represents a valuable biomaterial with interesting (mechanical) properties, which e.g., could be used after different ways of processing in bio-composite manufacturing as a basic or filler material for various fields of application. In addition to its good mechanical properties, the high lignin content is advantageous as it reduces molding, which would make such bio-composites applicable in building construction. Also, for bio-inspired technical puncture-resistant and damping materials, the hierarchical toughening structure of the coconut endocarp may serve as concept generator.

## Figures and Tables

**Table 1:** Morphological parameters of the different hierarchical levels of the coconut fruit. Given are mean values ± standard deviation. From hierarchical level H3, only the endocarp was considered. Abbreviations: number (n), thin to thick ratio (t:t ratio), vascular bundles (vb), sclereid cell matrix (scm), resolution (res), tracheids (t), sclereid cells (sc), mesocarp side (ms), centre (c), testa side (ts), cell wall (cw), width to length ratio (w:l ratio), primary cell wall layer (l1), secondary cell wall layers (l2), pits (p).

Hierarchical Level	Structure	n	Length	Width	Thickness	Others	Evaluated Data
**H0**	fruit	161	up to 25 [cm] ^1^	up to 20 [cm] ^1^	−	“ellipsoidal to broadly ovoid, indistinctly 3-angled” [Dransfield and Cooke, 1999]	
**H1**	−	−	−	−	−	“almost always 1 only, very large, with a narrow layer of homogeneous endosperm, and a large central cavity partially filled with fluid; embryo basal, opposite one of the endocarp pores” [Dransfield and Cooke, 1999]	
	pericarp	−	−	−	−	−	
**H2**	exocarp	−	−	−	0.015 [mm] ^2^	“smooth” [Dransfield and Cooke, 1999]; “a smooth, tough coat, of a brownish or grayish color” [Winton, 1901]	
	mesocarp	−	−	−	3–4 [cm] ^2^	“very thick and fibrous, dry” [Dransfield and Cooke, 1999]; “consists of a hard outer coat, but a few mm thick and a soft portion usually 3–4 cm thick on the sides and much thicker on the base. Imbedded in the mesocarp are numerous longitudinally arranged fibers, varying in size from slender hairs to large […] forms, 2–3 mm broad.” [Winton, 1901]	
	endocarp	10	116.5 ± 9.2 [mm]	92.8 ± 5.2 [mm]	thin: 2.5 ± 0.5 thick: 4.7 ± 0.6 [mm]	Shell with a prolate, spheroidal shape and varying thickness	digital calliper
**H3-1**	vb	−	−	−	−	diameter: 0.22 ± 0.14 [mm]	CT data (res: 42 µm) + CT Analyser
**H3-2**	scm					porosity: 0.033	CT data (res: 42 µm) + CT Analyser
**H4-1**	t	−	424.3 ± 263.3 [µm] (n = 6)	11.5 ± 3.6 [µm] (n = 80)	−	−	LM images (macerated cells) + ImageJ
**H4-2**	sc-ms	60	100.3 ± 21.5 [µm]	40.7 ± 9.2 [µm]	−	w:l ratio 0.42 ± 0.10	LM images (macerated cells) + ImageJ
	sc-c	60	115.6 ± 29.4 [µm]	31.3 ± 6.5 [µm]	−	w:l ratio 0.29 ± 0.09	LM images (macerated cells) + ImageJ
	sc-ts	60	125.2 ± 38.8 [µm]	25.4 ± 5.4 [µm]	−	w:l ratio 0.22 ± 0.09	LM images (macerated cells) + ImageJ
**H5-1**	cw (t)	30	−	−	1.7 ± 0.4 [µm]	scalariform pitting	SEM images + ImageJ
**H5-2**	cw (sc)	30	−	−	13.8 ± 2.9 [µm]	thickness in relation to cell radius 89.2 ± 4.3 [%]	SEM images + ImageJ
**H6-2**	l1 (sc)	24	−	−	2.0 ± 0.5 [µm] ^3^	−	SEM images + ImageJ
	l2 (sc)	145	−	−	253 ± 74 [nm]	−	SEM images + ImageJ
	p (sc)	414	2.5 ± 0.6 [µm]	1.8 ± 0.4 [µm]	−	bordered, ramiform pits	SEM images + ImageJ

^1^ data from Dransfield and Cooke [15]; ^2^ data from Winton [13]; ^3^ this value should be seen as a first order estimation and may be too high, including secondary cell wall layers and the middle lamella.

## Supplementary Materials

Supplementary materials are available online. Movie S1: CT-reconstruction of a coconut fruit (scan resolution: 208 µm), which was already sprouted. The hierarchical levels H0–H2 can be seen, though for reasons of clarity only the layers of H2 are named. The pericarp (H1) consists of the exocarp, mesocarp, and endocarp (H2). The seed (H1) comprises the endosperm, seedling, and testa (H2); the testa, a thin layer between the endocarp and endosperm, is not visible in this reconstruction. The video without text was kindly provided by the Shimadzu Corporation. Data S2: Raw data of the morphological parameters of the different hierarchical levels of the coconut.

## Appendix A. Protocol for Embedding the Endocarp in Acryl

All steps were done at room temperature and the sample was positioned on an orbital shaker (SHAKER-DOS 20S, neoLab Migge GmbH, Heidelberg, Germany), rotating with 120 rpm.

1. Transfer the sample to distilled water (duration: 30 min).
2. Transfer the sample to 70% EtOH (mixture of distilled water and EtOH (Ethanol 96% denaturated–T171.4, Carl Roth GmbH & Co. KG, Karlsruhe, Germany) at a ratio of three to seven) (duration: Overnight).
3. Transfer the sample to 70% EtOH (duration: 2 days).
4. Transfer the sample to 80% EtOH (duration: 1 day).
5. Transfer the sample to 80% EtOH (duration: 2 days).
6. Transfer the sample to 96% EtOH (duration: 1 day).
7. Transfer the sample to 96% EtOH (duration: 2 days).
8. Transfer the sample to 100% EtOH (duration: 1 day).
9. Transfer the sample to 100% EtOH (duration: 2 days).
10. Transfer the sample to 100% Xylene (#9713, Carl Roth GmbH & Co. KG, Germany) (duration: 1 day).
11. Transfer the sample to 100% Xylene (duration: 2 days).
12. Transfer the sample to mixture of Xylene and MMA (Methyl methacrylate, #8.00590.250, Merck KGaA, Darmstadt, Germany) at a ratio of one to one (duration: 7 days).
13. Transfer the sample to mixture of 100 mL MMA, 10 mL Di-n-butyl phthalate (Alfa Aesar, Thermo Fisher GmbH, Dreieich, Germany) and 1g Benzoyl peroxide (dried, #8.01641.1000, Merck KGaA, Germany) (duration: 3 days).
14. Transfer the sample to mixture of 100 mL MMA, 10 mL Di-n-butyl phthalate and 3g Benzoyl peroxide (dried) (duration: 14 days, until fully hardened).

Comment on this protocol: The embedding medium has not fully permeated the samples (partially air bubbles in vascular bundles observable, cell walls of stone cells not impregnated), but the embedding was sufficient for our purposes.
