# Supplementary material for: Hierarchical Structure of the Cocos nucifera (Coconut) Endocarp: Functional Morphology and its Influence on Fracture Toughness
Source: Molecules. 2020 Jan 6;25(1):223. doi: 10.3390/molecules25010223 (PMC6983247; doi:10.3390/molecules25010223)
Supplement: Supplementary file 1 [file molecules-25-00223-s001.zip › molecules-672669-Supplementary Materials 2.pdf]

Hierarchical structure of the *Cocos nucifera* (coconut) endocarp: Morphology and its influence on fracture toughness

Stefanie Schmier, Naoe Hosoda, Thomas Speck

Supplementary information 2

Raw data of the morphological parameters of the different hierarchical levels of the coconut endocarp

H2-Endocarp

no.endocarp	l1.mm	w1.mm	w2.mm	w3.mm	t.thick1.mm	t.thick2.mm	t.thick3.mm	t.thin1.mm	t.thin2.mm	t.thin3.mm
C1	102.1	92.0	91.2	95.0	5.24	5.36	5.31	1.76	2.19	1.78
C2	114.6	88.9	86.3	85.0	4.53	4.18	4.27	2.53	2.67	2.57
C3	122.0	87.4	88.5	82.4	3.97	3.77	3.75	2.17	1.95	2.07
C4	104.0	100.5	100.8	101.0	3.83	3.93	4.02	2.20	1.91	2.27
C5	126.8	96.8	94.3	94.5	4.00	4.28	3.97	2.19	2.05	2.01
C6	123.0	93.0	89.5	88.3	4.95	5.02	5.04	2.71	2.67	2.45
C7	106.1	89.5	90.0	88.8	4.46	4.43	4.30	3.81	3.63	3.41
C8	123.6	101.0	101.3	99.6	5.17	5.07	5.19	2.93	2.58	2.88
C9	119.8	91.4	90.3	90.6	5.34	5.42	5.09	2.59	2.64	2.74
C10	122.7	96.3	95.0	95.1	5.36	5.16	5.41	2.85	2.78	2.87

Abbreviations: l: endocarp length [mm]; w: endocarp width [mm]; t.thick: thickness thickest region of the cut surface [mm]; t.thin: thickness thinnest region of the cut surface [mm]

H3

Morphometry results of CT Analyser V.1.18.4.0 (Bruker microCT, Belgium)

Number of layers:	860
Lower vertical position:	4.1999E-002 [mm]
Upper vertical position:	3.6119E+001 [mm]
Pixel size:	4.1999E+001 [um]

Total VOI volume:	5.5667E+003 [mm³]
Object volume:	5.3810E+003 [mm³]
Total porosity:	3.3352E+000[%]

Diameter of vascular bundles (Structure separation):	2.1516E-001 [mm]
Standard deviation:	1.4118E-001 [mm]

Diameter of vascular bundles distribution:

Range	Volume	Percent volume in range
mm	mm³	%
4.1999E-002 - <1.2600E-001	5.3293E+001	27.9
1.2600E-001 - <2.1000E-001	6.9055E+001	36.1
2.1000E-001 - <2.9399E-001	2.5818E+001	13.5
2.9399E-001 - <3.7799E-001	1.9636E+001	10.3
3.7799E-001 - <4.6199E-001	1.2212E+001	6.4
4.6199E-001 - <5.4599E-001	4.6235E+000	2.4
5.4599E-001 - <6.2999E-001	3.3313E+000	1.7
6.2999E-001 - <7.1399E-001	8.9930E-001	0.5
7.1399E-001 - <7.9798E-001	1.0307E+000	0.5
7.9798E-001 - <8.8198E-001	9.8813E-001	0.5
8.8198E-001 - <9.6598E-001	3.6612E-001	0.2

H4-1

no.endocarp	position	l.complete	l.um	w.um
C1	mesocarp side	no	181.9	14.4
C1	mesocarp side	yes	869.2	15.0
C1	mesocarp side	yes	274.8	10.6
C1	mesocarp side	no	248.3	18.1
C1	mesocarp side	no	212.0	10.5
C1	mesocarp side	no	226.5	10.5
C1	mesocarp side	no	170.1	11.1
C1	mesocarp side	no	186.9	9.6
C1	mesocarp side	no	293.2	13.3
C1	centre	no	423.0	13.1
C1	centre	no	316.5	10.1
C1	centre	no	193.1	16.1
C1	centre	no	424.6	11.3
C1	centre	no	349.4	12.8
C1	centre	no	285.3	15.3
C1	centre	no	295.9	11.3
C1	centre	no	273.1	8.7
C1	centre	no	208.9	8.1
C1	centre	no	144.7	14.1
C1	testa side	no	401.8	7.5
C1	testa side	no	601.0	9.5
C1	testa side	no	419.6	12.0
C1	testa side	no	177.2	9.0
C1	testa side	no	347.0	11.7
C1	testa side	yes	290.1	11.1
C1	testa side	no	580.9	14.4
C1	testa side	no	619.4	19.5
C1	testa side	no	320.5	12.0
C1	testa side	no	340.3	14.9
C8	mesocarp side	no	605.6	22.6
C8	mesocarp side	no	259.3	12.0
C8	mesocarp side	no	587.5	23.6
C8	mesocarp side	no	548.3	24.5
C8	centre	no	82.7	10.0
C8	centre	no	269.9	10.6
C8	centre	no	420.4	13.7
C8	centre	no	237.8	11.0
C8	centre	no	400.9	13.5
C8	centre	no	143.0	8.0
C8	centre	no	159.2	13.4
C8	centre	no	181.2	13.0
C8	centre	no	226.1	9.7
C8	centre	yes	264.3	11.9

no.endocarp	position	l.complete	l.um	w.um
C8	testa side	yes	627.2	10.4
C8	testa side	no	144.7	9.5
C8	testa side	no	302.5	10.4
C8	testa side	no	185.9	11.9
C8	testa side	no	83.4	5.7
C8	testa side	no	194.2	12.4
C8	testa side	no	239.7	8.9
C8	testa side	no	87.1	6.5
C10	mesocarp side	yes	220.1	11.3
C10	mesocarp side	no	169.0	14.6
C10	mesocarp side	no	197.6	10.6
C10	mesocarp side	no	314.9	12.9
C10	mesocarp side	no	358.3	12.7
C10	mesocarp side	no	327.8	8.9
C10	mesocarp side	no	303.5	9.2
C10	mesocarp side	no	356.2	12.6
C10	mesocarp side	no	570.5	9.6
C10	mesocarp side	no	553.6	8.9
C10	centre	no	156.6	12.4
C10	centre	no	96.1	8.2
C10	centre	no	321.5	15.2
C10	centre	no	245.5	12.3
C10	centre	no	346.9	12.4
C10	centre	no	319.8	11.1
C10	centre	no	193.1	11.9
C10	centre	no	214.7	12.2
C10	centre	no	89.3	10.8
C10	testa side	no	111.6	8.1
C10	testa side	no	158.1	8.6
C10	testa side	no	189.2	7.0
C10	testa side	no	268.9	8.9
C10	testa side	no	155.4	5.8
C10	testa side	no	309.3	6.5
C10	testa side	no	250.8	7.4
C10	testa side	no	343.1	6.4
C10	testa side	no	316.6	10.4
C10	testa side	no	352.9	7.8

Abbreviations: l.complete: tracheid length intact, l.um: tracheid length [µm]; w.um: tracheid width [µm]

H4-2

no.endocarp	position	l.um	w.um	no.endocarp	position	l.um	w.um
C1	mesocarp side	89.1	48.1	C1	testa side	239.8	25.0
C1	mesocarp side	119.3	33.1	C1	testa side	104.1	27.8
C1	mesocarp side	107.7	52.9	C1	testa side	78.3	27.0
C1	mesocarp side	117.7	39.7	C1	testa side	106.5	26.3
C1	mesocarp side	98.9	30.2	C1	testa side	131.2	28.4
C1	mesocarp side	70.9	31.8	C1	testa side	89.6	24.7
C1	mesocarp side	89.8	36.0	C1	testa side	167.7	15.9
C1	mesocarp side	85.3	36.3	C1	testa side	181.2	24.1
C1	mesocarp side	89.1	47.9	C1	testa side	158.1	23.7
C1	mesocarp side	123.5	45.5	C1	testa side	170.4	24.1
C1	mesocarp side	116.2	43.6	C1	testa side	75.1	26.8
C1	mesocarp side	120.9	36.6	C1	testa side	159.3	18.2
C1	mesocarp side	95.7	47.0	C8	mesocarp side	134.7	58.3
C1	mesocarp side	76.8	28.0	C8	mesocarp side	124.0	39.7
C1	mesocarp side	76.4	24.6	C8	mesocarp side	105.0	55.6
C1	mesocarp side	115.6	48.4	C8	mesocarp side	140.6	38.8
C1	mesocarp side	91.6	31.5	C8	mesocarp side	136.0	37.5
C1	mesocarp side	75.4	41.7	C8	mesocarp side	130.6	58.1
C1	mesocarp side	94.6	37.3	C8	mesocarp side	97.5	31.6
C1	mesocarp side	82.0	29.1	C8	mesocarp side	124.1	64.4
C1	centre	143.6	29.5	C8	mesocarp side	72.5	31.9
C1	centre	124.8	32.1	C8	mesocarp side	76.6	41.9
C1	centre	79.9	35.8	C8	mesocarp side	130.4	32.0
C1	centre	139.9	18.3	C8	mesocarp side	100.9	42.8
C1	centre	112.1	24.3	C8	mesocarp side	90.8	48.3
C1	centre	117.7	30.8	C8	mesocarp side	141.4	45.9
C1	centre	75.8	22.4	C8	mesocarp side	75.9	26.2
C1	centre	127.0	39.4	C8	mesocarp side	113.6	61.1
C1	centre	127.1	28.4	C8	mesocarp side	109.4	43.9
C1	centre	82.8	28.8	C8	mesocarp side	74.6	37.5
C1	centre	143.5	33.3	C8	mesocarp side	108.4	48.1
C1	centre	162.1	33.5	C8	mesocarp side	129.5	45.9
C1	centre	126.2	26.1	C8	centre	119.6	33.0
C1	centre	114.2	40.6	C8	centre	126.2	52.2
C1	centre	133.8	25.5	C8	centre	91.8	36.5
C1	centre	145.8	32.8	C8	centre	97.5	32.1
C1	centre	87.7	19.8	C8	centre	130.1	27.9
C1	centre	79.7	34.7	C8	centre	172.7	21.7
C1	centre	74.0	27.0	C8	centre	168.4	40.7
C1	centre	176.5	43.6	C8	centre	99.9	40.2
C1	testa side	226.7	22.8	C8	centre	117.3	44.5
C1	testa side	172.5	20.2	C8	centre	135.9	31.9
C1	testa side	119.2	37.7	C8	centre	176.1	24.0
C1	testa side	110.3	22.3	C8	centre	137.3	28.5
C1	testa side	119.7	29.6	C8	centre	77.2	23.6
C1	testa side	201.5	38.1	C8	centre	93.5	33.1
C1	testa side	154.0	30.2	C8	centre	81.7	24.1
C1	testa side	154.2	22.4	C8	centre	88.7	34.5

no.endocarp	position	l.um	w.um
C8	centre	144.0	33.1
C8	centre	133.8	32.0
C8	centre	141.7	32.3
C8	centre	111.5	28.6
C8	testa side	72.1	25.9
C8	testa side	130.5	26.9
C8	testa side	161.2	20.9
C8	testa side	116.9	28.2
C8	testa side	171.5	26.6
C8	testa side	154.7	22.9
C8	testa side	83.5	22.6
C8	testa side	113.1	32.3
C8	testa side	100.7	29.8
C8	testa side	49.1	20.8
C8	testa side	91.0	21.2
C8	testa side	157.9	19.6
C8	testa side	138.8	14.4
C8	testa side	107.6	22.5
C8	testa side	85.1	16.3
C8	testa side	124.7	19.1
C8	testa side	101.6	25.1
C8	testa side	134.8	28.0
C8	testa side	124.6	19.2
C8	testa side	62.9	27.5
C10	mesocarp side	100.0	61.1
C10	mesocarp side	135.8	36.1
C10	mesocarp side	97.7	39.9
C10	mesocarp side	77.8	39.4
C10	mesocarp side	100.1	48.5
C10	mesocarp side	99.4	38.3
C10	mesocarp side	116.6	36.6
C10	mesocarp side	69.7	27.8
C10	mesocarp side	114.6	31.7
C10	mesocarp side	70.7	37.8
C10	mesocarp side	95.9	33.8
C10	mesocarp side	63.6	36.7
C10	mesocarp side	66.2	41.3
C10	mesocarp side	81.9	48.0
C10	mesocarp side	101.8	32.7
C10	mesocarp side	105.3	45.8
C10	mesocarp side	70.1	35.0
C10	mesocarp side	73.1	42.0
C10	mesocarp side	113.9	43.0
C10	mesocarp side	110.8	26.6

no.endocarp	position	l.um	w.um
C10	centre	106.9	32.4
C10	centre	78.0	33.5
C10	centre	78.8	25.4
C10	centre	152.8	24.5
C10	centre	108.7	33.5
C10	centre	100.2	43.5
C10	centre	129.3	30.8
C10	centre	71.6	26.6
C10	centre	157.5	29.1
C10	centre	87.3	24.5
C10	centre	104.6	34.7
C10	centre	73.5	37.9
C10	centre	102.7	27.0
C10	centre	98.5	30.7
C10	centre	147.3	29.8
C10	centre	149.3	27.5
C10	centre	102.6	29.0
C10	centre	89.2	37.6
C10	centre	91.7	22.5
C10	centre	90.1	34.2
C10	testa side	120.0	22.9
C10	testa side	163.0	24.9
C10	testa side	80.3	21.3
C10	testa side	81.8	29.8
C10	testa side	148.1	20.6
C10	testa side	152.0	21.4
C10	testa side	112.2	24.0
C10	testa side	133.3	38.4
C10	testa side	93.2	22.3
C10	testa side	83.7	27.8
C10	testa side	92.9	25.6
C10	testa side	95.2	34.0
C10	testa side	117.5	30.3
C10	testa side	119.2	21.4
C10	testa side	93.4	34.1
C10	testa side	133.8	37.2
C10	testa side	126.6	29.7
C10	testa side	157.6	23.4
C10	testa side	103.7	20.4
C10	testa side	101.6	26.6

Abbreviations: l.um: sclereid length [µm]; w.um: sclereid width [µm]

H5-1

no.endocarp	t1.um	t2.um	t3.um	r1.um	r2.um	r3.um
C1	1.1	0.9	1.1	4.2	4.1	4.8
C1	1.0	1.3	1.2	2.7	3.2	3.4
C1	1.7	1.7	1.5	4.5	4.5	4.2
C1	1.9	1.9	2.0	6.0	6.0	6.2
C1	1.1	1.1	1.2	4.4	3.8	4.1
C1	1.7	1.3	1.7	4.8	3.6	4.2
C1	2.2	2.2	2.5	7.0	7.0	7.1
C1	1.9	1.3	1.9	5.9	4.1	5.4
C1	2.6	3.4	2.5	9.2	9.7	7.5
C1	1.5	1.9	2.5	6.1	5.7	6.8
C8	2.0	1.7	2.4	7.1	5.5	7.7
C8	1.6	2.0	2.3	5.2	5.4	6.8
C8	1.3	1.2	1.3	4.3	3.9	4.4
C8	1.2	1.7	1.3	3.5	4.8	4.5
C8	2.0	1.8	1.4	5.7	7.0	5.5

no.endocarp	t1.um	t2.um	t3.um	r1.um	r2.um	r3.um
C8	1.4	2.2	1.5	5.0	5.5	4.8
C8	2.2	2.6	1.4	7.9	7.9	7.3
C8	1.8	1.7	1.9	9.5	8.7	10.5
C8	1.3	1.6	2.2	3.9	5.4	5.7
C8	1.9	2.3	2.0	6.9	8.4	7.8
C10	1.5	3.6	2.1	3.7	8.0	5.2
C10	2.2	1.3	1.0	9.3	6.8	5.0
C10	2.0	1.9	4.0	5.7	4.9	9.8
C10	1.5	1.5	1.1	6.4	5.7	4.6
C10	1.6	1.1	2.1	4.6	2.7	7.8
C10	1.4	1.1	1.3	5.5	5.2	5.3
C10	1.4	1.9	1.7	4.9	7.2	8.6
C10	1.2	1.2	1.0	5.3	5.1	5.9
C10	1.4	1.7	1.2	4.7	6.2	4.5
C10	1.4	1.2	1.4	5.4	4.7	5.1

Abbreviations: t: thickness tracheid cell wall [µm]; r: radius tracheid [µm]

H5-2

no.endocarp	no.cell	t1.um	t2.um	t3.um	r1.um	r2.um	r3.um
C1	S1	11.1	11.7	10.1	11.8	13.0	11.0
C1	S2	11.4	12.5	14.1	12.7	14.1	15.9
C1	S3	17.0	13.5	13.9	18.7	15.5	16.0
C1	S4	13.1	15.5	12.7	15.1	18.0	14.9
C1	S5	13.1	15.5	15.3	14.8	16.9	16.8
C1	S6	13.5	13.9	13.3	14.6	15.1	14.1
C1	S7	16.6	14.3	13.3	17.3	15.1	13.9
C1	S8	13.2	10.1	14.1	14.9	11.6	16.8
C1	S9	10.7	11.0	9.5	12.1	13.8	10.5
C1	S10	20.1	17.7	18.9	24.4	18.5	23.9
C8	S1	17.5	13.9	12.7	18.4	14.9	13.4
C8	S2	11.3	14.5	16.5	12.0	15.5	17.4
C8	S3	18.1	14.0	17.9	21.1	17.3	22.3
C8	S4	16.5	19.0	19.8	17.1	20.5	21.0
C8	S5	15.8	13.1	15.6	18.3	14.6	18.0
C8	S6	16.9	25.7	22.0	18.7	28.8	27.8
C8	S7	12.2	8.6	10.2	12.8	9.6	11.0
C8	S8	8.9	8.9	8.0	10.4	10.3	9.8
C8	S9	10.5	17.3	10.2	12.6	20.6	12.7
C8	S10	6.3	13.2	10.8	7.1	18.2	12.2
C10	S1	12.8	12.2	11.6	15.5	14.8	14.3
C10	S2	15.7	13.7	13.7	17.6	15.6	15.8
C10	S3	16.4	14.2	18.7	17.2	14.9	19.1
C10	S4	15.7	12.8	14.3	17.2	14.5	17.4
C10	S5	13.2	12.9	11.1	14.4	14.4	12.3
C10	S6	9.5	13.8	11.8	9.9	14.6	12.5
C10	S7	11.8	13.4	14.2	12.2	14.0	14.9
C10	S8	10.6	9.8	10.9	13.0	10.9	12.2
C10	S9	13.1	12.8	13.0	14.5	14.3	14.7
C10	S10	16.9	23.0	13.4	18.0	26.8	14.5

Abbreviations: no.cell: ; t: thickness sclereid cell wall [µm]; r: radius sclereid [µm]

H6-2

Primary cell wall layer (l1)

no.endocarp	no.cell	t.um	no.endocarp	no.cell	t.um	no.endocarp	no.cell	t.um
C1	S1	2.55	C8	S1	2.23	C10	S1	2.48
C1	S2	NA	C8	S2	1.88	C10	S2	1.88
C1	S3	2.34	C8	S3	2.06	C10	S3	1.43
C1	S4	1.75	C8	S4	1.55	C10	S4	2.30
C1	S5	1.70	C8	S5	1.10	C10	S5	2.74
C1	S6	NA	C8	S6	NA	C10	S6	1.57
C1	S7	2.09	C8	S7	NA	C10	S7	NA
C1	S8	2.31	C8	S8	2.16	C10	S8	1.86
C1	S9	1.51	C8	S9	1.22	C10	S9	NA
C1	S10	3.17	C8	S10	2.40	C10	S10	2.65

Secondary cell wall layers (l2)

no.endocarp	no.cell	t.um	no.endocarp	no.cell	t.um	no.endocarp	no.cell	t.um	no.endocarp	no.cell	t.um
C1	S1	0.25	C1	S7	0.24	C8	S6	0.18	C10	S8	0.24
C1	S1	0.31	C1	S10	0.27	C8	S6	0.19	C10	S8	0.23
C1	S1	0.21	C1	S10	0.29	C8	S6	0.23	C10	S8	0.28
C1	S1	0.26	C1	S10	0.24	C8	S6	0.22	C10	S8	0.25
C1	S1	0.19	C1	S10	0.33	C8	S6	0.17	C10	S8	0.20
C1	S1	0.20	C1	S10	0.35	C8	S6	0.17	C10	S8	0.14
C1	S1	0.27	C1	S10	0.29	C8	S6	0.18	C10	S8	0.17
C1	S1	0.14	C1	S10	0.34	C8	S6	0.16	C10	S8	0.17
C1	S1	0.19	C1	S10	0.33	C8	S6	0.17	C10	S9	0.23
C1	S1	0.14	C1	S10	0.27	C8	S6	0.14	C10	S9	0.26
C1	S3	0.34	C1	S10	0.29	C8	S10	0.17	C10	S9	0.25
C1	S3	0.48	C8	S3	0.28	C8	S10	0.17	C10	S9	0.26
C1	S3	0.47	C8	S3	0.34	C8	S10	0.21	C10	S9	0.19
C1	S3	0.31	C8	S3	0.37	C8	S10	0.16	C10	S9	0.21
C1	S3	0.29	C8	S3	0.43	C8	S10	0.17	C10	S9	0.22
C1	S3	0.30	C8	S3	0.32	C8	S10	0.17	C10	S9	0.22
C1	S3	0.28	C8	S3	0.34	C8	S10	0.14	C10	S9	0.17
C1	S3	0.30	C8	S3	0.29	C8	S10	0.17	C10	S9	0.20
C1	S3	0.37	C8	S3	0.41	C8	S10	0.15	C10	S10	0.23
C1	S3	0.28	C8	S4	0.34	C8	S10	0.21	C10	S10	0.32
C1	S4	0.21	C8	S4	0.30	C10	S1	0.34	C10	S10	0.33
C1	S4	0.21	C8	S4	0.26	C10	S1	0.24	C10	S10	0.21
C1	S4	0.23	C8	S4	0.24	C10	S1	0.31	C10	S10	0.23
C1	S4	0.28	C8	S4	0.24	C10	S1	0.27	C10	S10	0.33
C1	S4	0.20	C8	S4	0.28	C10	S1	0.27	C10	S10	0.43
C1	S4	0.20	C8	S4	0.35	C10	S1	0.25	C10	S10	0.37
C1	S4	0.22	C8	S4	0.34	C10	S1	0.21	C10	S10	0.47
C1	S4	0.21	C8	S4	0.33	C10	S1	0.22	C10	S10	0.46
C1	S4	0.15	C8	S4	0.38	C10	S3	0.28			
C1	S4	0.20	C8	S5	0.22	C10	S3	0.24			
C1	S7	0.19	C8	S5	0.24	C10	S3	0.21			
C1	S7	0.22	C8	S5	0.26	C10	S3	0.31			
C1	S7	0.18	C8	S5	0.21	C10	S3	0.26			
C1	S7	0.22	C8	S5	0.20	C10	S3	0.22			
C1	S7	0.29	C8	S5	0.25	C10	S3	0.24			
C1	S7	0.29	C8	S5	0.21	C10	S3	0.24			
C1	S7	0.32	C8	S5	0.16	C10	S3	0.18			
C1	S7	0.25	C8	S5	0.18	C10	S3	0.21			
C1	S7	0.32	C8	S5	0.22	C10	S8	0.22			

Pits

no.endocarp	no.cell	l.um	w.um
C1	S11	2.15	1.84
C1	S11	2.47	1.70
C1	S11	2.52	1.71
C1	S11	2.54	1.51
C1	S11	1.71	1.16
C1	S11	2.25	1.57
C1	S11	2.44	1.23
C1	S11	2.25	1.53
C1	S11	2.41	1.86
C1	S11	3.10	1.80
C1	S11	2.38	1.40
C1	S11	1.91	1.63
C1	S11	2.35	1.74
C1	S11	2.07	1.89
C1	S11	2.33	1.61
C1	S11	2.30	1.70
C1	S11	3.99	2.02
C1	S11	2.26	1.64
C1	S11	1.85	1.59
C1	S11	2.43	1.60
C1	S11	2.35	1.77
C1	S11	2.13	1.53
C1	S11	2.62	2.17
C1	S12	2.61	2.05
C1	S12	2.71	1.78
C1	S12	2.79	1.79
C1	S12	1.50	1.01
C1	S12	3.27	2.40
C1	S12	1.70	1.57
C1	S12	2.45	2.09
C1	S12	1.71	1.18
C1	S12	1.97	1.43
C1	S12	2.37	1.88
C1	S12	2.21	1.77
C1	S12	3.00	1.94
C1	S12	3.10	2.17
C1	S12	1.94	1.77
C1	S12	1.93	1.30
C1	S12	2.07	1.92
C1	S12	2.50	1.70
C1	S12	2.48	1.77
C1	S12	2.37	1.99
C1	S12	3.11	2.18
C1	S12	2.87	2.04
C1	S13	2.09	1.89
C1	S13	1.81	1.68
C1	S13	1.92	1.45
C1	S13	1.81	1.41
C1	S13	2.14	1.76
C1	S13	1.78	1.36
C1	S13	2.40	2.00
C1	S13	2.09	1.42
C1	S13	2.48	1.74

no.endocarp	no.cell	l.um	w.um
C1	S13	1.78	1.31
C1	S13	1.18	1.18
C1	S13	2.14	1.90
C1	S13	3.36	1.60
C1	S13	3.32	2.54
C1	S13	2.29	1.72
C1	S13	2.57	1.68
C1	S13	2.93	2.17
C1	S13	3.58	2.44
C1	S14	3.27	2.28
C1	S14	2.13	1.88
C1	S14	2.52	1.75
C1	S14	3.25	2.44
C1	S14	1.46	1.16
C1	S14	3.11	2.06
C1	S14	1.98	1.04
C1	S14	3.02	1.32
C1	S14	4.43	1.93
C1	S14	1.18	0.75
C1	S14	2.49	1.47
C1	S14	2.64	2.20
C1	S14	1.38	0.84
C1	S14	3.89	2.32
C1	S14	2.71	1.92
C1	S14	3.06	1.92
C1	S14	2.10	1.68
C1	S14	2.83	1.50
C1	S14	2.54	2.19
C1	S15	3.31	2.04
C1	S15	2.69	2.06
C1	S15	1.76	1.32
C1	S15	3.68	2.40
C1	S15	2.20	2.14
C1	S15	1.77	1.53
C1	S15	2.51	2.10
C1	S15	3.04	2.25
C1	S15	2.73	1.90
C1	S15	2.61	1.77
C1	S15	3.53	2.81
C1	S15	3.77	2.04
C1	S15	2.41	1.76
C1	S15	2.43	2.19
C1	S15	2.57	2.20
C1	S15	2.75	1.62
C1	S15	3.93	2.31
C1	S15	3.45	2.09
C1	S15	3.08	1.76
C1	S15	2.19	1.55
C1	S15	3.12	1.61
C1	S15	2.48	1.84
C1	S15	2.18	1.76
C1	S15	2.44	1.91
C1	S15	3.21	1.97

no.endocarp	no.cell	l.um	w.um
C1	S16	1.50	1.37
C1	S16	1.69	1.56
C1	S16	2.08	2.04
C1	S16	2.73	2.45
C1	S16	1.50	1.33
C1	S16	2.53	2.08
C1	S16	3.00	2.53
C1	S16	2.06	1.91
C1	S16	2.36	2.24
C1	S16	1.38	1.06
C1	S16	2.52	1.87
C1	S16	3.64	2.53
C1	S16	3.16	1.90
C1	S16	2.76	1.95
C1	S16	1.56	1.37
C1	S16	3.19	2.14
C1	S16	3.14	2.06
C1	S16	3.56	2.28
C1	S16	2.76	1.77
C1	S16	2.92	1.77
C1	S16	2.57	1.88

no.endocarp	no.cell	l.um	w.um
C8	S11	3.07	2.55
C8	S11	2.02	1.80
C8	S11	2.22	1.92
C8	S11	2.74	2.01
C8	S11	1.77	1.36
C8	S11	2.21	1.74
C8	S11	2.52	1.78
C8	S11	2.70	2.02
C8	S11	1.88	1.51
C8	S11	1.72	1.68
C8	S11	2.34	1.84
C8	S11	3.29	2.15
C8	S11	2.70	2.33
C8	S11	2.85	2.50
C8	S11	2.56	1.91
C8	S11	1.94	1.64
C8	S11	2.60	1.90
C8	S11	2.86	2.54
C8	S11	3.53	2.95
C8	S11	2.67	1.98
C8	S11	2.74	2.42
C8	S11	1.88	1.46
C8	S11	2.56	1.79
C8	S11	2.85	2.00
C8	S11	2.98	2.02
C8	S11	1.99	1.43
C8	S11	2.16	1.66
C8	S11	2.08	1.84
C8	S11	2.08	1.68
C8	S11	2.49	1.82
C8	S11	2.82	2.35
C8	S11	2.32	2.17
C8	S11	3.04	2.16
C8	S11	2.47	2.28
C8	S11	4.04	2.89
C8	S11	2.74	2.68
C8	S11	3.09	2.64
C8	S12	1.77	1.38
C8	S12	2.10	1.77
C8	S12	1.91	1.47
C8	S12	2.41	1.80
C8	S12	2.56	1.87
C8	S12	2.27	1.65
C8	S12	1.92	1.63
C8	S12	2.02	1.45
C8	S12	2.90	2.16
C8	S12	1.81	1.69
C8	S12	2.32	2.11
C8	S12	1.70	1.62
C8	S12	2.67	1.81
C8	S12	2.47	1.88
C8	S12	2.95	1.78
C8	S12	3.30	1.75
C8	S13	2.33	1.75

no.endocarp	no.cell	l.um	w.um
C8	S13	2.68	1.79
C8	S13	2.40	1.45
C8	S13	3.35	1.95
C8	S13	1.96	1.34
C8	S13	2.60	2.03
C8	S13	2.09	1.80
C8	S13	1.54	1.31
C8	S13	1.71	1.67
C8	S13	1.83	1.59
C8	S13	3.08	1.80
C8	S13	3.58	2.55
C8	S13	2.95	1.93
C8	S14	1.68	1.43
C8	S14	3.87	2.54
C8	S14	2.29	1.94
C8	S14	2.12	1.73
C8	S14	1.35	1.27
C8	S14	1.65	1.39
C8	S14	2.06	1.61
C8	S14	1.90	1.67
C8	S14	2.72	1.89
C8	S14	2.21	1.45
C8	S14	1.87	1.03
C8	S14	2.01	1.77
C8	S14	2.80	2.37
C8	S14	2.27	1.87
C8	S14	2.18	2.00
C8	S14	4.24	2.18
C8	S14	2.13	1.47
C8	S14	2.11	1.54
C8	S14	3.40	1.64
C8	S14	2.44	1.74
C8	S14	2.47	1.58
C8	S14	2.48	1.78
C8	S14	2.39	2.24
C8	S14	2.05	1.83
C8	S14	1.22	0.98
C8	S14	2.73	2.09
C8	S14	2.29	1.35
C8	S14	2.42	1.77
C8	S14	2.63	2.13
C8	S14	2.46	1.98
C8	S14	2.45	1.51
C8	S14	2.48	1.88
C8	S14	2.74	2.31
C8	S14	2.44	2.01
C8	S14	2.47	2.25
C8	S14	3.00	2.19
C8	S14	3.24	2.78
C8	S14	1.95	1.46
C8	S14	3.42	2.35
C8	S15	2.40	1.70
C8	S15	1.36	1.29
C8	S15	1.25	0.96

no.endocarp	no.cell	l.um	w.um
C8	S15	1.51	1.28
C8	S15	2.47	2.03
C8	S15	2.56	1.92
C8	S15	2.12	1.75
C8	S15	2.25	2.00
C8	S15	2.33	1.72
C8	S15	2.69	1.99
C8	S15	1.42	1.07
C8	S15	2.78	1.76
C8	S15	2.76	1.65
C8	S15	2.07	1.22
C8	S15	2.31	2.19
C8	S15	2.46	1.72
C8	S15	2.21	1.85
C8	S15	1.92	1.30
C8	S15	2.47	1.54
C8	S16	2.20	1.72
C8	S16	2.79	2.44
C8	S16	3.60	2.71
C8	S16	2.43	1.97
C8	S16	2.43	1.76
C8	S16	2.13	1.95
C8	S16	1.92	1.47
C8	S16	2.63	1.82
C8	S16	1.29	0.84
C8	S16	1.85	1.40
C8	S16	2.42	1.66
C8	S16	2.04	1.62
C8	S16	2.82	1.92

no.endocarp	no.cell	l.um	w.um
C10	S11	1.68	1.45
C10	S11	2.53	1.79
C10	S11	2.13	1.63
C10	S11	2.79	1.77
C10	S11	1.45	1.17
C10	S11	2.48	1.99
C10	S11	1.60	1.38
C10	S11	2.67	1.95
C10	S11	3.60	2.15
C10	S11	2.23	2.12
C10	S11	3.06	2.73
C10	S11	2.96	2.33
C10	S11	3.78	2.06
C10	S11	3.11	2.12
C10	S11	3.38	2.26
C10	S11	2.70	2.00
C10	S11	1.95	1.63
C10	S11	2.13	1.88
C10	S11	1.84	1.75
C10	S11	4.14	2.10
C10	S11	2.59	2.54
C10	S11	2.27	1.99
C10	S12	2.51	2.49
C10	S12	2.11	2.04
C10	S12	2.19	1.58
C10	S12	2.35	2.05
C10	S12	2.35	1.71
C10	S12	3.06	1.68
C10	S12	3.26	1.86
C10	S12	2.44	1.96
C10	S12	3.15	2.02
C10	S12	2.10	1.75
C10	S12	2.50	1.90
C10	S12	2.65	1.82
C10	S12	2.66	2.42
C10	S12	2.92	1.44
C10	S12	2.04	1.88
C10	S12	1.79	1.60
C10	S12	2.83	2.15
C10	S12	1.89	1.62
C10	S12	1.89	1.70
C10	S12	3.38	1.80
C10	S12	2.73	2.09
C10	S12	2.78	1.93
C10	S12	2.67	1.93
C10	S12	2.30	1.68
C10	S13	1.59	1.29
C10	S13	2.26	1.83
C10	S13	2.35	1.67
C10	S13	2.45	1.78
C10	S13	1.96	1.54
C10	S13	2.40	1.83
C10	S13	2.43	1.54
C10	S13	1.51	1.23
C10	S13	2.69	2.10

no.endocarp	no.cell	l.um	w.um
C10	S13	1.81	1.25
C10	S13	1.80	1.44
C10	S13	1.31	1.05
C10	S13	1.94	1.68
C10	S13	2.19	2.05
C10	S13	2.16	2.11
C10	S14	2.47	1.92
C10	S14	2.50	1.52
C10	S14	2.52	1.40
C10	S14	2.77	1.35
C10	S14	3.04	1.62
C10	S14	2.35	1.46
C10	S14	5.02	1.76
C10	S14	5.17	2.04
C10	S14	2.07	1.09
C10	S14	1.84	1.09
C10	S14	2.98	1.46
C10	S14	3.22	1.78
C10	S14	1.92	1.14
C10	S14	3.45	2.06
C10	S14	2.61	1.75
C10	S14	2.73	1.45
C10	S14	2.05	1.60
C10	S14	2.42	1.36
C10	S14	1.97	1.19
C10	S14	2.25	1.50
C10	S14	1.69	1.34
C10	S14	3.09	1.84
C10	S14	2.46	1.20
C10	S14	1.79	1.10
C10	S14	2.05	1.61
C10	S14	2.13	1.41
C10	S14	1.88	1.52
C10	S14	3.06	1.63
C10	S14	2.56	1.42
C10	S14	1.99	1.83
C10	S14	1.77	1.20
C10	S14	1.63	1.28
C10	S14	2.27	1.28
C10	S14	2.96	1.85
C10	S14	1.84	1.54
C10	S15	2.75	1.87
C10	S15	2.78	1.50
C10	S15	2.24	1.65
C10	S15	2.19	1.92
C10	S15	2.50	1.58
C10	S15	2.60	1.56
C10	S15	2.18	1.43
C10	S15	1.78	1.44
C10	S15	2.50	1.10
C10	S15	1.63	1.19
C10	S15	2.18	1.22
C10	S15	2.18	1.59
C10	S15	2.39	1.27
C10	S15	2.56	1.56

no.endocarp	no.cell	l.um	w.um
C10	S15	2.24	1.00
C10	S15	2.56	1.47
C10	S15	2.81	1.67
C10	S15	1.95	1.37
C10	S15	2.02	1.66
C10	S15	2.54	1.43
C10	S15	2.45	1.43
C10	S15	2.97	1.57
C10	S15	2.81	1.67
C10	S15	2.91	1.33
C10	S15	2.16	1.59
C10	S15	2.59	0.98
C10	S15	3.59	1.84
C10	S15	1.65	1.06
C10	S15	1.98	1.34
C10	S16	3.89	2.17
C10	S16	3.09	2.07
C10	S16	2.90	2.06
C10	S16	2.14	1.74
C10	S16	2.56	2.10
C10	S16	2.94	2.51
C10	S16	2.95	2.16
C10	S16	2.21	2.03
C10	S16	2.46	1.57
C10	S16	2.69	1.87
C10	S16	3.16	2.27
C10	S16	3.08	2.74
C10	S16	1.15	1.10
C10	S16	4.15	2.46
C10	S16	2.06	1.41
C10	S16	1.82	1.23
C10	S16	3.30	2.08
C10	S16	3.03	2.02
C10	S16	2.08	1.67
C10	S16	2.23	1.83
C10	S16	3.01	1.93
C10	S16	3.22	2.18
C10	S16	1.71	1.33
C10	S16	3.67	2.67
C10	S16	2.64	1.57
